# Using statutory health insurance data to evaluate non-response in a cross-sectional study on depression among patients with diabetes in Germany

**DOI:** 10.1093/ije/dyz278

**Published:** 2020-01-28

**Authors:** Ute Linnenkamp, Veronika Gontscharuk, Manuela Brüne, Nadezda Chernyak, Tatjana Kvitkina, Werner Arend, Annett Fiege, Imke Schmitz-Losem, Johannes Kruse, Silvia M A A Evers, Mickaël Hiligsmann, Barbara Hoffmann, Silke Andrich, Andrea Icks

**Affiliations:** 1 Institute for Health Services Research and Health Economics, German Diabetes Center (DDZ), Leibniz Center for Diabetes Research at Heinrich-Heine University Düsseldorf, Düsseldorf, Germany; 2 German Center for Diabetes Research (DZD), Neuherberg, Germany; 3 Department of Health Services Research, CAPHRI Care and Public Health Research Institute, Maastricht University, Maastricht, The Netherlands; 4 Institute for Health Services Research and Health Economics, Centre for Health and Society, Faculty of Medicine, Heinrich-Heine University Düsseldorf, Düsseldorf, Germany; 5 pronova BKK, Statutory Health Insurance, Ludwigshafen, Germany; 6 Clinic for Psychosomatic and Psychotherapy, University Clinic Gießen, Gießen, Germany; 7 Trimbos Institute, Netherlands Institute of Mental Health and Addiction, Utrecht, The Netherlands; 8 Institute for Occupational, Social and Environmental Medicine, Centre for Health and Society, Faculty of Medicine, Heinrich-Heine University Düsseldorf, Düsseldorf, Germany

**Keywords:** Diabetes, non-response, health survey, depression, quality of life

## Abstract

**Background:**

Low response rates do not indicate poor representativeness of study populations if non-response occurs completely at random. A non-response analysis can help to investigate whether non-response is a potential source for bias within a study.

**Methods:**

A cross-sectional survey among a random sample of a health insurance population with diabetes (*n* = 3642, 58.9% male, mean age 65.7 years), assessing depression in diabetes, was conducted in 2013 in Germany. Health insurance data were available for responders and non-responders to assess non-response bias. The response rate was 51.1%. Odds ratios (ORs) for responses to the survey were calculated using logistic regression taking into consideration the depression diagnosis as well as age, sex, antihyperglycaemic medication, medication utilization, hospital admission and other comorbidities (from health insurance data).

**Results:**

Responders and non-responders did not differ in the depression diagnosis [OR 0.99, confidence interval (CI) 0.82–1.2]. Regardless of age and sex, treatment with insulin only (OR 1.73, CI 1.36–2.21), treatment with oral antihyperglycaemic drugs (OAD) only (OR 1.77, CI 1.49–2.09), treatment with both insulin and OAD (OR 1.91, CI 1.51–2.43) and higher general medication utilization (1.29, 1.10–1.51) were associated with responding to the survey.

**Conclusion:**

We found differences in age, sex, diabetes treatment and medication utilization between responders and non-responders, which might bias the results. However, responders and non-responders did not differ in their depression status, which is the focus of the DiaDec study. Our analysis may serve as an example for conducting non-response analyses using health insurance data.

**Key Messages**

- Unexpectedly, the responders did not differ from the non-responders in having a history of depression diagnosis in a survey among patients with diabetes.
- Responders and non-responders differed in other characteristics, such as age and sex, with older persons being more likely to respond than younger persons and females being less likely to respond than males.
- We could use a comprehensive set of sociodemographic and health-related data for both responders and non-responders to analyse the representativeness of responders with large explanatory power.

## Introduction

Traditionally, the representativeness of surveys in social and health-care science is evaluated on the response rates.^1–3^ However, within studies using survey data, it is not expected to receive a complete response from every individual invited to participate, even though a complete response is the primary goal.^4^ In the past decades, response rates worldwide, especially those in western countries, have declined.^5–8^ Recent research suggests that the representativeness of a study is not inevitably dependent on its response rate.^1^^,^^9^^,^^10^ If the non-responses to the survey occurred completely at random, a low response rate does not necessarily indicate poor representativeness of the study population.^1^ Unfortunately, the non-responses rarely occur completely at random. With non-responses, we might obtain biased results with regard to prevalence or incidence and with regard to associations between health outcomes and risk factors. Differences between responders and non-responders have been observed with respect to socio-economic and demographic characteristics as well as variations in health status. Non-responders are, for example, more likely to live alone,^5^^,^^6^ to be less educated, to have a poorer lifestyle and a worse health status than are responders.^6^ However, little evidence is available on the difference between responders and non-responders regarding the variables of primary interest in health surveys because key measures are usually not available for non-responders. By using health insurance data, we had the unique opportunity to assess differences between responders and non-responders, focusing on a variable that is the main outcome of our survey: depression status.

This paper focuses on the comparison of responders with non-responders within the project ‘Quality of life, disability, health care utilization and costs in patients with diabetes: The role of depression’ (DiaDec). The aims of the DiaDec study are to estimate the prevalence of depression symptoms among patients with diabetes and to evaluate the association between comorbid depression status and health-care utilization/costs as well as health-related quality of life (HRQoL). With this paper, we aim to answer the following three questions: (1) Is the depression status different between responders and non-responders? (2) Are sociodemographic characteristics related to non-response in the DiaDec study? (3) Are diabetes treatment, comorbidities and health-care utilization associated with response status?

## Methods

### Design of the DiaDec study

A detailed description of the study design and methods is available from Kvitkina *et al*.^11^ In short, we conducted a cross-sectional nine-page postal survey in a random sample of a German statutory health insurance (SHI) population with diabetes and later linked the data on an individual level to longitudinal SHI data. The baseline survey was conducted during 2013. All responders who gave consent and the SHI data on health-care utilization patterns and health-care costs that were available for the period between 12 months before and 12 months after the baseline survey were included. The questionnaire assessed sociodemographic variables, depression symptoms, HRQoL and diabetes-specific distress. By linking the survey data with longitudinal SHI data, the association between depression status, HRQoL and health-care utilization/costs in the year before and after the survey were investigated.

### Study population and recruitment procedure

**Figure 1.**
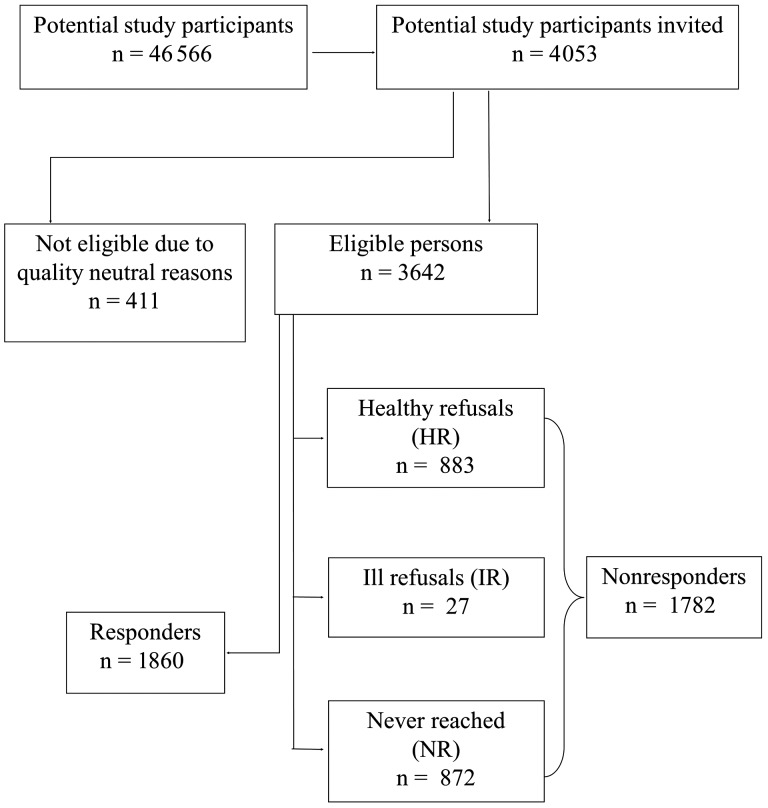
Flow chart for selection of study participants.

Of the 636 451 individuals insured by pronova Betriebskrankenkassen (BKK), a national statutory health insurance company, 46 566 individuals had diabetes and were thus potential study participants in the DiaDec study (Fig. 1). Eligibility was based on a diagnosis of diabetes in the SHI data as defined and validated by the criteria of Hauner *et al*., that have been used in previous studies.^12^^,^^13^ From the 46 566 potential study participants, a random sample of 4053 subjects were selected and invited to participate in the study. The postal recruitment procedure was carried out in 3 months: March, May and August 2013. The recruitment materials included a cover letter, which included information on the study, the questionnaire, a data protection and declaration of consent to use the SHI data and a stamped, addressed return envelope. If the individual did not respond within 3–8 weeks, a reminder letter was sent. If the individual did not respond to the reminder letter we attempted to contact the individual by telephone 3–7 weeks later at least twice. People were also contacted if the questionnaire was not filled in completely or if they forgot to return the signed consent form and only sent the questionnaire back.

### Description of the variables included in the non-response analysis

For the non-response analysis, the SHI provided individual-level information for responders and non-responders for the following variables within the year prior to the baseline survey (2012): age (categorized into four groups: <50, 50–59, 60–69, 70–80 years of age), sex, treatment with insulin (yes or no), use of oral antihyperglycaemic drugs (OAD) (yes or no), depression diagnosis (yes or no), number of hospitalization cases, number of medications prescribed (based on the count of pharmaceutical registration numbers in the year prior to the baseline survey) and other comorbidities. Insulin usage was defined as at least one record of an Anatomical Therapeutic Chemical Classification System (ATC) code ‘A10A’ covering ‘insulins and analogues’. The use of OAD was defined as at least one record of an ATC code ‘A10B’ covering ‘blood glucose lowering drugs, excluding insulins’, though records of ‘A10BX04’ (exenatide) and ‘A10BX07’ (liraglutide) were excluded. The two variables focusing on antihyperglycaemic medication for diabetes were combined into one variable with four characteristics: treatment with insulin only, treatment with OAD only, treatment with a combination of insulin and OAD and no treatment with insulin or OAD. A diagnosis of depression (yes or no) was defined as at least one record of the ICD-10 code ‘F32’ (major depressive disorder, single episode), ‘F33’ (major depressive disorder, recurrent) or ‘F34.1’ (dysthymic disorder). Comorbidities were based on the morbidity-oriented risk compensation scheme.^14^ This system covers 80 either ‘severe’ or ‘costly and chronic’ diseases that are structured in a system of hierarchical groups. We defined comorbidities as the number of diagnoses the patient had of the 80 included diagnoses. Information on health-care utilization was provided by using the number of hospital admissions and the number of medications prescribed (based on the number of different pharmacy product numbers).

### Statistical methods

The available data were used to conduct an analysis of non-response to evaluate whether non-response occurred completely at random or if the non-responders differed systematically from the responders of the survey.

Of all the potential study participants, except for those not eligible for quality neutral reasons, those who returned the questionnaire and signed consent form were regarded as responders to our survey. All individuals who did not return the questionnaire, the signed consent form or both documents were regarded as non-responders. We further classified the non-responders according to the classification system by Slattery *et al*. and Stang *et al*. as never reached (NR), ill refusals (IR) and healthy refusals (HR).^15^^,^^16^ The NR are the individuals who never answered the invitation and were not reachable in follow-up telephone calls since they changed their phone number or the telephone was constantly busy. Individuals who refused to participate either due to medical conditions, such as living in an elderly care home, being hospitalized, being in rehabilitation, being dependent on nursing care, or being ill were referred to as IRs. The individuals who refused to participate due to reasons not related to medical conditions were regarded as HRs. The reason for refusal was reported by either the contacted person or a relative of that person either in the questionnaire or during the follow-up telephone call.

The analyses proceeded in three steps. First, we estimated the contact, cooperation and response rates in accordance with the method suggested by Slattery *et al*. and Stang *et al*.^15^^,^^16^ The contact rate describes the proportion of potential study participants we were able to establish contact with regardless of the participants’ eligibility (i.e. the number of responders, HR, IR and persons not eligible for quality neutral reasons divided by the number of potential study participants invited (Supplementary Figure S1, available as Supplementary data at *IJE* online). The cooperation rate indicates the rate of completed and returned surveys among all contacts by eligible persons (i.e. the number of responders divided by the number of responders and refusals). Thus, the cooperation rate expresses the number of individuals willing to participate in relation to the number of eligible persons who could be reached. The response rate provides the proportion of eligible persons who completed and returned the survey (i.e. the number of responders divided by the number of eligible persons). We have listed the exact formulas for calculating the rates in the Supplementary material.

Bivariate comparisons were performed using a Mann–Whitney U test for quantitative variables and a Pearson’s chi-square test (alternatively, Fisher’s exact test when possible) for categorical variables. The significance level was set to α = 0.05. We used logistic regression to determine whether the following variables/terms were associated with response status: age class, sex, interaction between age group and sex, antihyperglycaemic medication, depression diagnosis and comorbidities and health-care utilization (medication and hospital admissions). We dichotomized the following variables based on the median into two categories: comorbidities (≤3, >3), number of medication prescriptions (≤22, >22) and hospital admissions (0, ≥1).

We showed the reliability and reproducibility of our analysis in two ways. First, we compared the original estimated odds ratios (ORs) with the resampled ORs obtained through permutations. In doing so, the group membership becomes arbitrary if we assume that there is no difference in the variables between the responders and non-responders, as any subject from one group could be from the other group. We randomly divided the original sample into two random groups of 1860 and 1782 persons 999 times. We labelled these groups as dummy responders and non-responders and carried out the above-mentioned logistic regression for each of these 999 samples (Supplementary Figure S2, available as Supplementary data at *IJE* online). Resampled ORs mimic the null hypothesis of no difference existing between the responders and non-responders for a given data set. Second, we compared the original ORs with the ORs based on the 999 bootstrap samples (Supplementary Figure S3, available as Supplementary data at *IJE* online). Bootstrap samples were created by selecting 1860 observations for non-responders and 1782 observations for responders from the original groups. One person was selected randomly and returned to the dataset before the selection process was repeated, which suggests that one person could have been chosen multiple times. Bootstrap ORs illustrate how stable the estimated ORs are.

### Ethics statement

Ethical approval was obtained from the ethics committee of the Heinrich-Heine University Düsseldorf and is available under the study reference 3762.

## Results

We were informed that of the 4053 randomly selected persons identified as potential study participants invited to participate in the DiaDec study, 411 were not suitable to receive a questionnaire due to quality neutral reasons (Fig. 1). In total, 3642 eligible persons insured with the SHI and diagnosed with diabetes received a questionnaire and a consent form. Of these persons, 1860 were responders and 1782 were non-responders. Eight hundred and seventy-two (48.9%) non-responders were classified as NR, 27 persons (1.5%) were considered IR and 883 (49.6%) were considered HR.

### Contact, response and cooperation rates

Within the DiaDec study, the overall contact rate was 78.5%, the cooperation rate was 67.1% and the overall response rate was 51.1%.

**Figure 2.**
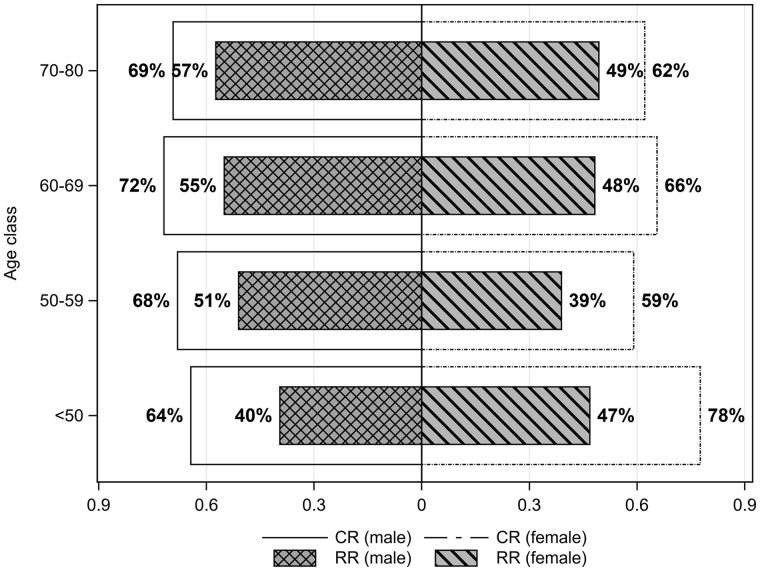
Response rates (RR) and cooperation rates (CR) stratified for age and sex.

The response rates were higher for females than for males in the <50 years age group and higher for males than for females in the >50 years age groups (Fig. 2). The cooperation and response rates showed similar patterns, as they were both higher for females than for males in the <50 years age group and higher for males than for females in the >50 years age groups.

### Description of the whole sample and comparison of responders and non-responders

The mean age of the sample was 65.7 years, and 41.1% of the individuals were female (Table 1). In the year prior to the survey, 16.3% of the individuals had a diagnosis of depression. Approximately half of the individuals were treated with OAD only, ∼14% were treated with a combination of OAD and insulin, ∼13% were treated with insulin only and ∼24% were not treated with either insulin or OAD. The mean number of comorbidities in the sample in the year prior to the survey was 3.4. The mean number of prescribed medications in the year prior to the survey was 26.3, and the mean number of hospital admissions was 0.4. The responders were slightly older than the non-responders. The responders and non-responders differed in almost all of the characteristics. No difference was found in the diagnosis of depression, which is one of the main variables of interest in the DiaDec study.

**Table 1.** Description of the whole DiaDec sample

Variable	Whole sample	Responders	Non-responders	*P*-value^a^
	*n* = 3642	*n* = 1860	*n* = 1782	
Age				
Mean, SD (range)	65.7, 10.6 (17.0 –79.9)	66.5, 9.9 (17.0–79.9)	64.9, 11.2 (17.5–79.9)	0.0002
Median (IQR)	67.9 (59.8 -74.0)	68.7 (60.9–74.2)	67.1 (58.3–73.7)	
Age class, *n* (%)				
<50	321 (8.81%)	135 (7.26%)	186 (10.44%)	0.0001
50–59	618 (16.97%)	288 (15.48%)	330 (18.52%)	
60–69	1130 (31.03%)	591 (31.77%)	539 (30.25%)	
70–80	1573 (43.19%)	846 (45.48%)	727 (40.80%)	
Sex, *n* (%)				
Female	1496 (41.1%)	707 (38.0%)	789 (44.3%)	0.0001
Antihyperglycaemic drugs, *n* (%)				
Insulin only	466 (12.8%)	256 (13.8%)	210 (11.8%)	< 0.0001
Oral antihyperglycaemic drugs only	1785 (49.0%)	959 (51.6%)	826 (46.4%)	
Insulin and oral antihyperglycaemic drugs	525 (14.4%)	310 (16.7%)	215 (12.1%)	
Depression diagnosis, *n* (%)	595 (16.3%)	303 (16.3%)	292 (16.4%)	0.9642
Comorbidities				
Mean, SD (range)	3.4, 2.1 (0.0–17.0)	3.7, 2.2 (0.0–17.0)	3.2, 1.9 (0.0–16.0)	< 0.0001
Median (IQR)	3.0 (2.0 - 4.0)	3.0 (2.0–5.0)	3.0 (2.0–4.0)	
Health care utilization				
Medication utilization				
Mean, SD (range)	26.3, 19.9 (0.0–360.0)	28.7, 20.6 (0.0–360.0)	23.7, 18.9 (0.0–359.0)	< 0.0001
Median (IQR)	22.0 (13.0–35.0)	24.0 (15.0–38.5)	20.0 (11.0–31.0)	
Hospital admissions,				
Mean, SD (range)	0.4, 1.0 (0.0–16.0)	0.5, 1.1 (0.0–16.0)	0.4, 0.9 (0.0–13.0)	0.0009
Median (IQR)	0.0 (0.0–1.0)	0.0 (0.0–1.0)	0.0 (0.0–0.0)	

^a^ *P*-values are reported for differences between responders and non-responders.

IQR, inter quartile range; SD, standard deviation.

### Results of the logistic regression

Table 2 presents the results of the logistic regression. All variables included in the model were associated with participation in our study except the depression diagnosis, number of comorbidities and hospital admissions. Higher medication utilization [OR 1.29, confidence interval (CI) 1.10–1.51], treatment with insulin only (OR 1.73, CI 1.36–2.21), treatment with OAD only (OR 1.77, CI 1.49–2.09) and treatment with both insulin and OAD (OR 1.91, CI 1.51–2.43) rates were associated with responses to the mailed survey in our study, whereas being female was associated with a lower likelihood of responding to the survey for those aged 50–59 years (OR 0.60, CI 0.43–0.84), for those aged 60–69 years (OR 0.74, CI 0.58–0.94), and for those aged 70–80 years (OR 0.72, CI 0.58–0.88) (Supplementary Table S1, available as Supplementary data at *IJE* online). The interaction between age group and sex was visible only for the group of individuals <50 years old (β = 0.68 with *P*-value 0.01) (Table 2). Although a younger age and being female were associated with non-responses, females <50 years old seemed to participate slightly more often than males of the same age group (Supplementary Table S1, available as Supplementary data at *IJE* online).

**Table 2.** Results of logistic regression analysis to assess factors associated with participation in the DiaDec study; *n* = 3642. Reference group: age class (70–80), male, no antihyperglycaemic drugs, no depression diagnosis, comorbidities ≤3, medical utilization ≤22, no hospital admission. *R*^2^ of the logistic regression, 0.0401

Variable	β	*P*-value	Odds ratio	95 % Confidence intervals	
				Lower	Upper
Age class^a^					
Age class (<50 vs 70–80)	−0.6285	0.0001	0.53^b^	0.39	0.73
Age class (50–59 vs 70–80)	−0.2123	0.0906	0.81^b^	0.63	1.03
Age class (60–69 vs 70–80)	−0.0678	0.5215	0.93^b^	0.76	1.15
Gender^a^					
Sex (female vs male)	−0.3334	0.0014	0.72^c^	0.58	0.88
Antihyperglycaemic drugs					
Insulin only vs no medication	0.5500	<0.0001	1.73	1.36	2.21
Oral antihyperglycaemic drugs only vs no medication	0.5696	<0.0001	1.77	1.49	2.09
Insulin and oral antihyperglycaemic drugs vs no medication	0.6491	<0.0001	1.91	1.51	2.43
Comorbidities					
Depression diagnosis (yes vs no)	−0.0070	0.9422	0.99	0.82	1.20
Comorbidities (>3 vs ≤3)	0.1588	0.0508	1.17	1.00	1.37
Health-care utilization					
Medication utilization (>22 vs ≤22 )	0.2525	0.0017	1.29	1.10	1.51
Hospital admissions (≥1 vs 0)	0.0845	0.3068	1.09	0.93	1.28

a Interaction terms for age class and sex were included in the model: sex (female) x age class (<50 years): ß = 0.6800, *P* = 0.0093; Sex (female) x age class (50–59 years): ß = −0.1807, *P* = 0.3683; sex (female) x age class (60–69 years): ß = 0.0276, *P* = 0.8639. The full set of ORs related to gender x age class group comparison is shown in Table 1 Supplementary Table S1.

b Age comparison for male participants.

c Sex comparison for participants within oldest age class (70–80).

Although several of the considered variables were associated with responses, some of the ORs were small to moderate, e.g. those for hospital admission and medical utilization. The (pseudo) *R*^2^ was 0.04, indicating a weak relationship between the considered variables and response status.

The results of the original logistic regression seem to be reliable and reproducible (Supplementary Figures S2 and S3, available as Supplementary data at *IJE* online).

## Discussion

The most important result of our analysis was that depression status was not associated with responses to our survey, but the opposite trend could be assumed. Since we assessed the depression diagnoses obtained 1 year prior to the survey, the individuals identified as having depression may not have been experiencing depression symptoms at the time of the survey. Another explanation might be that our study specifically focuses on persons with diabetes and depression, which may be of interest to people who hope to contribute towards the improvement of care of people in a similar situation in the future by participating in the survey.

We found that sociodemographic characteristics were related to non-responses: older age was associated with a higher likelihood of responding to the survey, whereas being female was associated with a lower likelihood of responding to the survey. Nevertheless, young women were more likely to participate in the survey due to a possible interaction between age and sex.

Furthermore, we found antihyperglycaemic medication, health-care utilization and higher medication utilization to be associated with responses to the mailed survey. Response was higher in those taking medications compared with those not taking medications, irrespective of the type of medication. However, comparing the impact of the different types of antihyperglycaemic medications showed that there were no associations with the response status, suggesting that the type of medication was not important. Age and sex as well as medical utilization seem to be of moderate importance with regard to participation in the DiaDec study.

### Comparison with other studies

The overall response rate was 51.1% in the DiaDec study, which is within the range reported by a study using a similar methodology (linking questionnaire data with SHI data). The study among patients with coronary heart disease of a German SHI was conducted in 2013 and reported a considerably lower response rate of 32.6%.^17^ Lin *et al*. used a comparable population survey for health maintenance organization enrolees with diabetes in the United States in their study conducted in 2003, and they reported a response rate of 62.0%.^18^ Pilot surveys of the European Health Examination Survey conducted between 2009 and 2012 in 12 countries (Czech Republic, Finland, Germany, Greece, Italy, Malta, The Netherlands, Norway, Poland, Portugal, Slovakia, The United Kingdom) showed that obtaining participation rates >50% is possible but requires well-planned recruitment strategies and a large amount of effort by the investigators.^19^

Studies on non-response rates differ to a large extent in their design, primary aim, mode of comparison, characteristics compared and various other aspects. Several studies found that sex and age impact the non-response rate ^20–24^ and that women are less likely to participate in health surveys than men, which is similar to the results of our study. No clear pattern has evolved regarding the sex–age interaction. Education, socio-economic status and marital status also impact non-response rates to health surveys.^5^^,^^6^^,^^20^^,^^21^^,^^25^^,^^26^ However, this relationship could not be investigated within the analysis of non-responders in the DiaDec study due to missing information in our health-care-based data set. Instead, we used, for the first time, other characteristics, such as diabetes treatment, depression status, health-care utilization, number of comorbidities and higher medication utilization, to assess the differences between responders and non-responders.

### Limitations and strengths of our study

Our analysis has some limitations. For example, we were not able to gather information on the socio-economic status of non-responders; we did not have information on the migration status of the invited participants, which might also be a determinant of non-responses.^23^ Furthermore, we could only analyse the presence of depression in general but could not differentiate between the different types of depression, which might have differed between the responders and non-responders. Last, we did not assess the exact reason for individuals not responding to our survey, which would have helped to obtain a better understanding of the reasons why people did not respond.

As suggested by Kho *et al*., we collected a minimum dataset of key variables of all the people identified to be eligible to participate in our study by using SHI data.^27^ Therefore, we were able to analyse the whole sample of invited persons not only with respect to the demographic characteristics but also with respect to relevant characteristics of primary interest in our health survey. Furthermore, we could directly match the data of the responders and non-responders of our survey with another data set including data that was not self-reported but externally validated and provided the same information for responders as for non-responders.^28^

Since there are considerable differences in the prevalence of chronic diseases in different SHIs in Germany,^29^ it cannot be claimed that the results of the DiaDec study are representative of the whole German population, as the sample is only representative of one SHI. However, the SHI is a national SHI.

## Conclusion

It is often a concern that responders and non-responders may differ in their health status, introducing a non-response bias to the study results. The aim of the DiaDec study was to analyse how patients with diabetes and depression differ from patients with diabetes but without depression in various aspects, e.g. health-care utilization or health-related quality of life. Since depression is one of the main variables of interest within the DiaDec study, it is of major importance for the interpretation of the results in the DiaDec study that responders and non-responders did not differ with regard to the depression diagnoses. Future research studies should assess the reasons for non-response, at least in a subsample of non-responders, to determine how to minimize the potential bias in this kind of research study. The thorough assessment of non-response bias in this study is essential for the appropriate interpretation of the possible differences between the DiaDec study participants with and without depression and may serve as an example of how to assess non-response bias by using SHI data.

## Supplementary Material

dyz278_Supplementary_Data
