# Supplementary material for: Using statutory health insurance data to evaluate non-response in a cross-sectional study on depression among patients with diabetes in Germany
Source: Int J Epidemiol. 2020 Jan 28;49(2):629–37. doi: 10.1093/ije/dyz278 (PMC7266537; doi:10.1093/ije/dyz278)
Supplement: dyz278_Supplementary_Data [file dyz278_supplementary_data.zip › dyz278-suppl_data/ije-2019-02-0216-File006.docx]

Supplementary Figure S1: *Formulas to calculate contact, cooperation and response rates*

$Contact Rate = \frac{responders+refusals+ persons not eligible due to quality neutral reasons}{potential study participants invited}$

$= \frac{1860+910+411}{4053}=78.5 \%$

$Cooperation rate= \frac{responders}{responders+ refusals}$

$= \frac{1860}{1860+910}=$ *67.1 %*

$Response rate = \frac{\mathrm{responders}}{eligible persons}$

$= \frac{1860}{3642}=$ *51.1%*
