# Supplementary material for: Using statutory health insurance data to evaluate non-response in a cross-sectional study on depression among patients with diabetes in Germany
Source: Int J Epidemiol. 2020 Jan 28;49(2):629–37. doi: 10.1093/ije/dyz278 (PMC7266537; doi:10.1093/ije/dyz278)
Supplement: dyz278_Supplementary_Data [file dyz278_supplementary_data.zip › dyz278-suppl_data/ije-2019-02-0216-File009.docx]

Supplementary Table S1: Comparison of responders and nonresponders in the DiaDec study by age class and sex

| **Age class and sex group comparison** | **P-value** | **Odds ratio** | **95 % Confidence Intervals** | |
| --- | --- | --- | --- | --- |
|  |  |  | **Lower** | **Upper** |
| Sex at age class |  |  |  |  |
| Sex (female vs. male) at age class (<50) | 0.1488 | 1.41 | 0.88 | 2.26 |
| Sex (female vs. male) at age class (50-59) | 0.0029 | 0.60 | 0.43 | 0.84 |
| Sex (female vs. male) at age class (60-69) | 0.0137 | 0.74 | 0.58 | 0.94 |
| Sex (female vs. male) at age class (70-80) | 0.0014 | 0.72 | 0.58 | 0.88 |
| Age class at sex |  |  |  |  |
| Age class (<50 vs. 70-80) for male | 0.0001 | 0.53 | 0.39 | 0.73 |
| Age class (50-59 vs. 70-80) for male | 0.0906 | 0.81 | 0.63 | 1.03 |
| Age class (60-69 vs. 70-80) for male | 0.5215 | 0.93 | 0.76 | 1.15 |
| Age class (<50 vs. 60-69) for male | 0.0007 | 0.57 | 0.41 | 0.79 |
| Age class (50-59 vs. 60-69) for male | 0.2641 | 0.87 | 0.67 | 1.12 |
| Age class (<50 vs. 50-59) for male | 0.0187 | 0.66 | 0.47 | 0.93 |
| Age class (<50 vs. 70-80) for female | 0.8070 | 1.05 | 0.70 | 1.59 |
| Age class (50-59 vs. 70-80) for female | 0.0136 | 0.68 | 0.49 | 0.92 |
| Age class (60-69 vs. 70-80) for female | 0.7439 | 0.96 | 0.76 | 1.22 |
| Age class (<50 vs. 60-69) for female | 0.6735 | 1.10 | 0.72 | 1.68 |
| Age class (50-59 vs. 60-69) for female | 0.0358 | 0.70 | 0.51 | 0.98 |
| Age class (<50 vs. 50-59) for female | 0.0630 | 1.56 | 0.98 | 2.49 |
| Different age class and sex |  |  |  |  |
| Female (<50) vs. male (70-80) | 0.1775 | 0.75 | 0.50 | 1.14 |
| Female (50-59) vs. male (70-80) | <.0001 | 0.48 | 0.36 | 0.66 |
| Female (60-69) vs. male (70-80) | 0.0018 | 0.69 | 0.54 | 0.87 |
| Female (<50) vs. male (60-69) | 0.3105 | 0.81 | 0.53 | 1.22 |
| Female (50-59) vs. male (60-69) | <.0001 | 0.52 | 0.38 | 0.71 |
| Female (70-80) vs. male (60-69) | 0.0162 | 0.77 | 0.62 | 0.95 |
| Female (<50) vs. male (50-59) | 0.7521 | 0.93 | 0.61 | 1.44 |
| Female (60-69) vs. male (50-59) | 0.2515 | 0.85 | 0.65 | 1.12 |
| Female (70-80) vs. male (50-59) | 0.3493 | 0.89 | 0.69 | 1.14 |
| Female (50-59) vs. male (<50) | 0.6253 | 0.91 | 0.61 | 1.34 |
| Female (60-79) vs. male (<50) | 0.1432 | 1.29 | 0.92 | 1.82 |
| Female (70-80) vs. male (<50) | 0.0748 | 1.34 | 0.97 | 1.86 |
