# Supplementary figures and images for: Using statutory health insurance data to evaluate non-response in a cross-sectional study on depression among patients with diabetes in Germany
Source: Int J Epidemiol. 2020 Jan 28;49(2):629–37. doi: 10.1093/ije/dyz278 (PMC7266537; doi:10.1093/ije/dyz278)

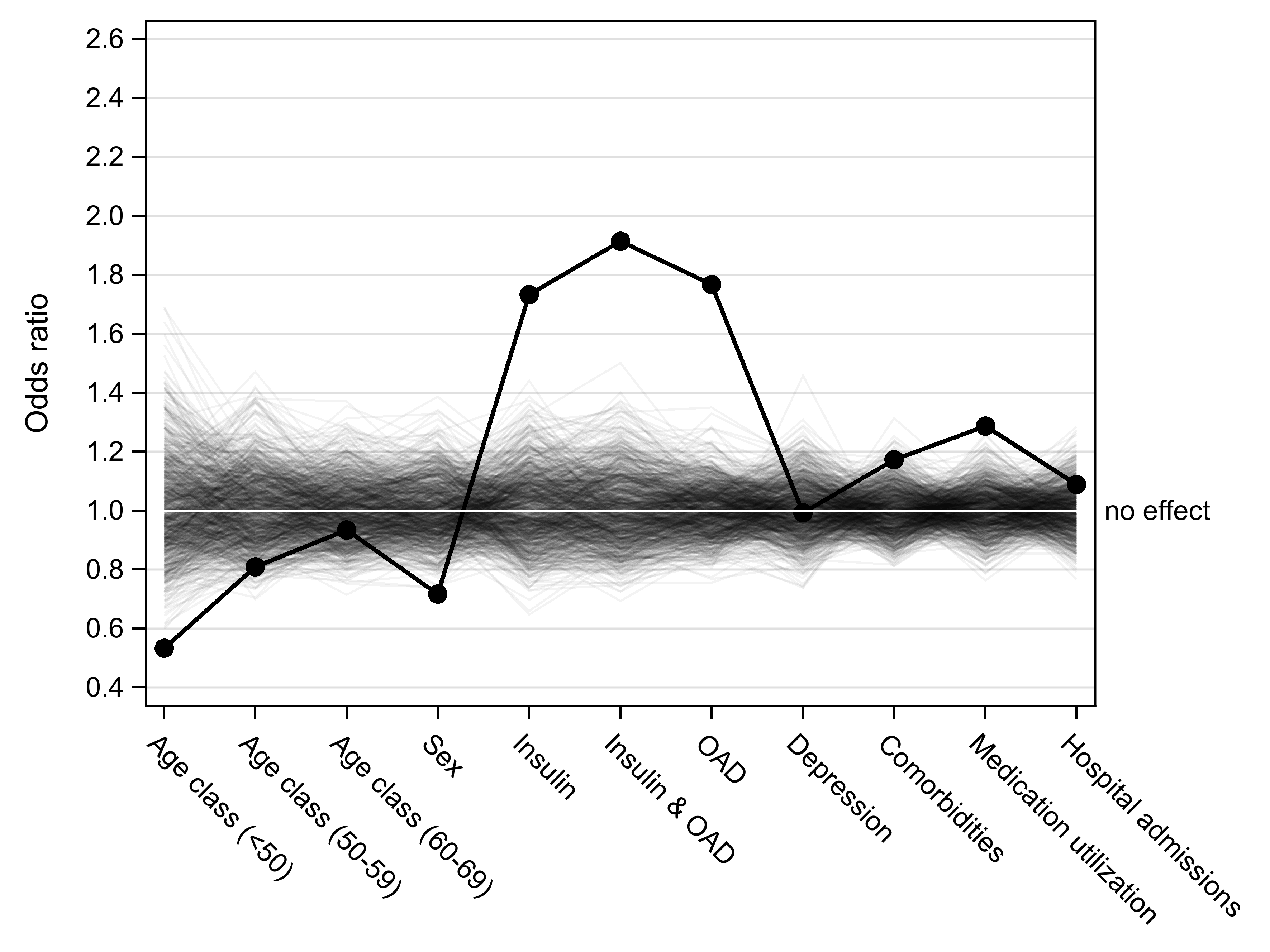

Supplement: dyz278_Supplementary_Data [file dyz278_supplementary_data.zip › dyz278-suppl_data/ije-2019-02-0216-File007.tiff]

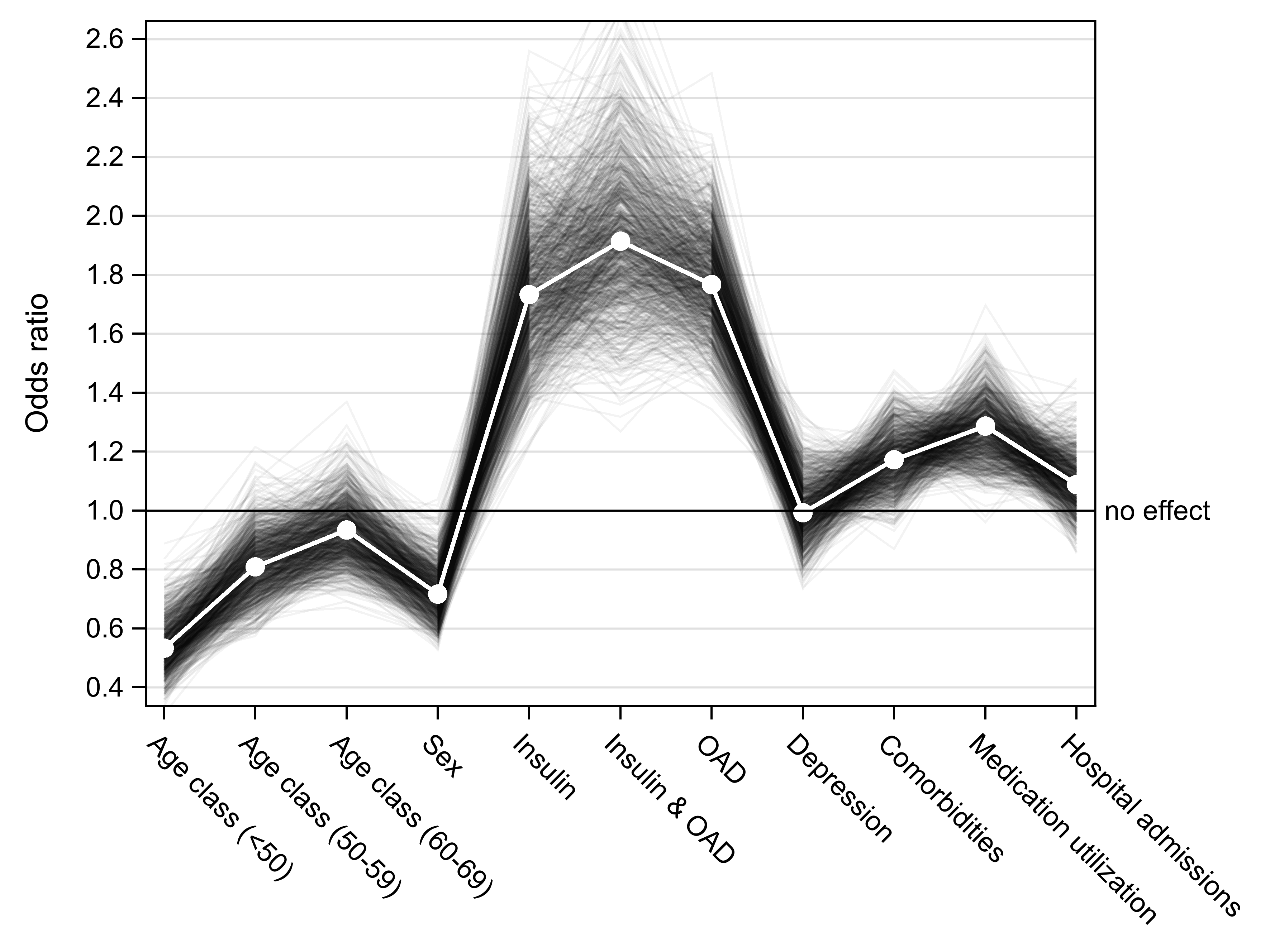

Supplement: dyz278_Supplementary_Data [file dyz278_supplementary_data.zip › dyz278-suppl_data/ije-2019-02-0216-File008.tiff]
